# Case report: Novel *ACTN4* variant of uncertain significance in a pediatric case of steroid-resistant nephrotic syndrome requesting kidney transplantation

**DOI:** 10.3389/fneph.2024.1375538

**Published:** 2025-01-31

**Authors:** Ignacio Alarcón, Carolina Peralta, Francisco Cammarata-Scalisi, Maykol Araya Castillo, Francisco Cano, Angélica Rojo, María Luisa Ceballos, Paola Krall

**Affiliations:** ^1^ Escuela de Medicina, Facultad de Medicina, Universidad de Chile, Santiago, Chile; ^2^ Servicio de Pediatría, Hospital de Antofagasta, Antofagasta, Chile; ^3^ Laboratorio Clínico, Hospital de Antofagasta, Antofagasta, Chile; ^4^ Departamento de Pediatría y Cirugía Infantil Oriente, Facultad de Medicina, Universidad de Chile, Santiago, Chile; ^5^ Unidad de Nefrología, Hospital Luis Calvo Mackenna, Santiago, Chile; ^6^ Instituto de Medicina, Facultad de Medicina, Universidad Austral de Chile, Valdivia, Chile

**Keywords:** steroid-resistant nephrotic syndrome, variant of uncertain significance, ACTN4, pediatric kidney transplantation, focal and segmental glomerulosclerosis, recurrence risk

## Abstract

**Background:**

Steroid-resistant nephrotic syndrome (SRNS) is a rare kidney disease commonly characterized histopathologically by focal and segmental glomerulosclerosis (FSGS) or minimal change disease. One-third of SRNS-FSGS cases are attributed to a genetic cause ultimately leading to end-stage kidney disease (ESKD) during childhood or adulthood. *ACTN4* variants, although rare, typically manifest in early adulthood as SRNS-FSGS with autosomal dominant inheritance pattern and are associated with variable progression toward ESKD.

**Case–diagnosis/treatment:**

A 10-year-old Chilean male patient, born to a complicated pregnancy without any history of prenatal care, was incidentally found to have mild proteinuria during pre-surgery analysis. He was diagnosed with nephrotic syndrome and treatment with prednisone was started, but 12 months later, he persisted with hyperlipidemia, hypoalbuminemia, and proteinuria. Within a few weeks, proteinuria rapidly increased, and a kidney biopsy exhibited FSGS features. At the age of 12, he reached ESKD and initiated peritoneal dialysis, experiencing an episode of posterior reversible encephalopathy syndrome. Exome sequencing identified a novel variant of uncertain significance (VUS), *ACTN4* c.625_633del that predicted the in-frame deletion p.L209_E211del in a highly conserved functional domain. He requested to be considered for kidney transplantation and the VUS in *ACTN4* was re-analyzed to assess potential risks, resulting in a reclassification as likely pathogenic (PM1+PM2+PM4 criteria). At 14 years old, he received a deceased donor kidney allograft without recurrence during the subsequent 5 months.

**Conclusions:**

Identifying VUS is a recurring challenge in routine clinical genetics, particularly for patients with rare diseases or atypical phenotypes in underrepresented populations. This case underscores the benefit of timely genetic diagnosis taking into account the patient’s request. VUS reassessment becomes more relevant when considering a kidney transplant not only as an appropriate procedure, but as the therapy of choice, especially considering the patient’s history of complications with variable long-term consequences.

## Introduction

Steroid-resistant nephrotic syndrome (SRNS) is a kidney disease with annual incidence estimated at 1/390,000 that is characterized by a failed response to steroid treatment in patients, evident after a 4–6-week course of daily prednisone (1). Clinically, SRNS presents with proteinuria, hypoalbuminemia, edema, and hyperlipidemia. Histopathologically, the biopsy reveals the presence of focal and segmental glomerulosclerosis (FSGS) or minimal change disease (MCD). A subgroup of SRNS patients might achieve remission, but up to 50% that start in childhood reach end-stage kidney disease (ESKD) within 10-15 years (2). Once the SRNS diagnosis is established, it is essential to elucidate the underlying mechanism to propose clinical management. However, this condition exhibits different etiologies, attributing one-third of all cases to genes related to the podocytes and the glomerular basement membrane. To date, more than 60 genes have been linked to SRNS-FSGS, with variants most frequently identified in key genes such as nephrin (*NPHS1*) and podocin (*NPHS2*), integral membrane proteins of the slit diaphragm; Wilms tumor protein (*WT1*), a transcription tumor suppressor protein; phospholipase C epsilon (*PLCE1*), involved in podocyte proliferation and differentiation; and alpha-actinin-4 (*ACTN4*), which regulates the podocyte cytoskeleton; among other genes (3, 4).

*ACTN4* gene is located on chromosome 19q13.2 and comprises 21 exons, which encode a protein consisting of 911 amino acids (5). ACTN4 is an actin crosslinking protein that structurally consists of a long rod domain that connects the amino terminal functional actin-binding domain (ABD) and the carboxyl calcium binding motif and presents in antiparallel homodimers (6). Of note, the ABD contains two calponin-homology (CH1 and CH2) domains that harbor the majority of *ACTN4* variants described in association with SRNS-FSGS (7).

To date, at least 20 pathogenic or likely pathogenic *ACTN4* variants have been identified. Patients carrying these allele variations are unique and typically manifest the adult-onset form of SRNS (8). On the other hand, Varsome (https://varsome.com/) reports over 100 variants of unknown significance (VUS) in *ACTN4*, which, according to the Standards and Guidelines for the interpretation of Sequence Variants by the American College of Medical Genetics (ACMG), should not be used solely for clinical decision-making. In these cases, and in the absence of proactive updates, the recommendation is to perform segregation analysis within the patient family, whenever possible, or to perform periodic consultations to determine whether there have been modifications or reclassification of any VUS that would allow for therapeutic decision-making (9).

In this study, we present a Chilean male patient who was carrier of a novel variant in *ACTN4*, initially classified as VUS, that was re-evaluated given the rapid progression to ESKD in order to guide decisions regarding kidney transplantation.

## Case report

**Figure 1:**
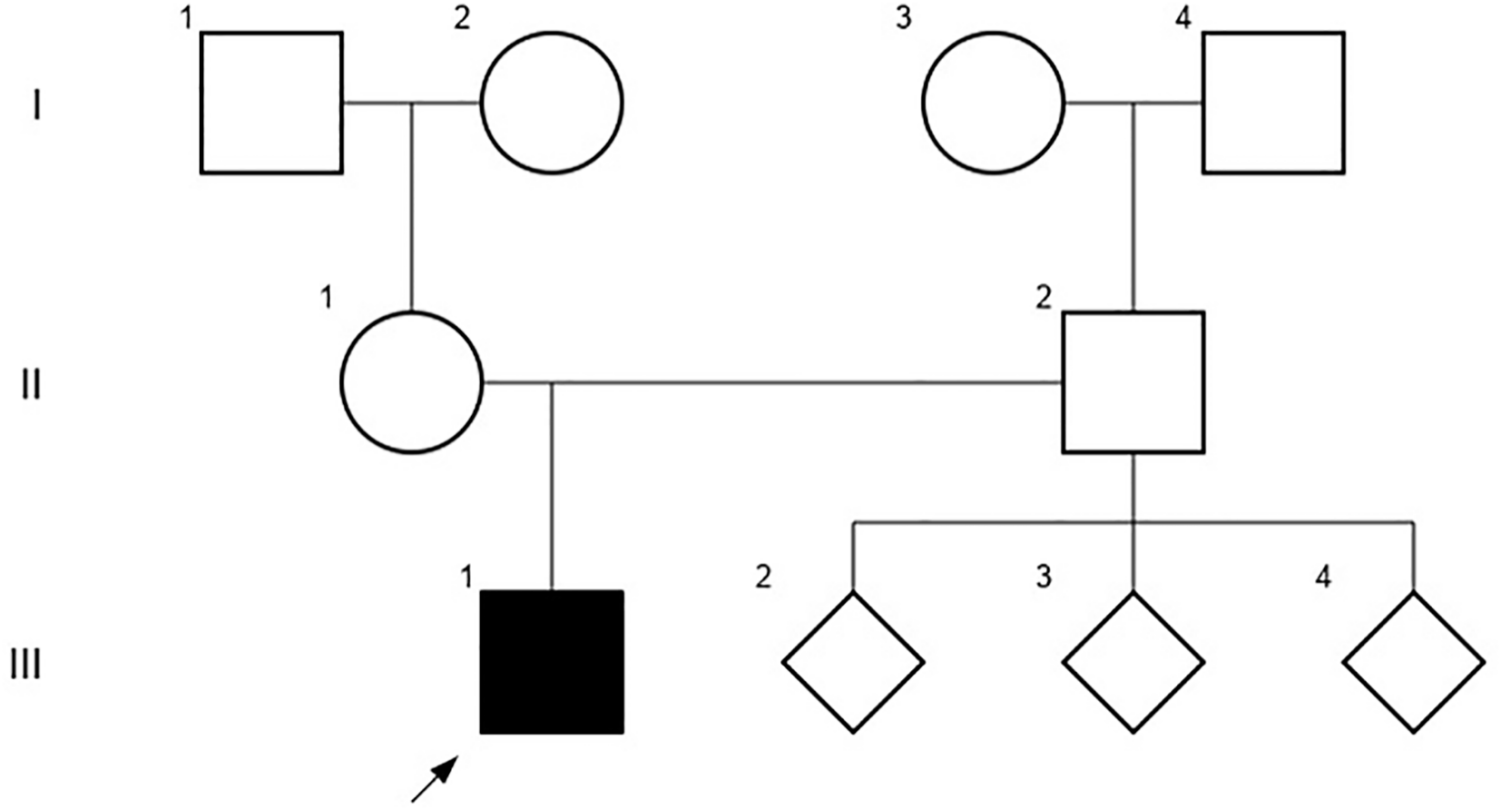
The patient´s pedigree chart, including third-degree relatives that did not document history of kidney disease at the time of the study.

A 10-year-old Chilean male patient, born to a cocaine-abuse complicated pregnancy without any history of prenatal care, was found to have mild proteinuria (spot dipstick +1) on a urine analysis. This finding occurred in the context of planning a relapsed bilateral cryptorchidism surgery. The grandmother was mentioned to be his legal tutor since he was 12 months old and she could not provide information about his gestational age and birth weight. He did not receive breastfeeding and his parents were non-consanguineous. He had a history of hypothyroidism and asthma, both under treatment, during early childhood. No significant familial background of kidney disease was documented, and the patient had three healthy paternal siblings (**Figure 1**).

At the time of the first nephrological evaluation, he did not present psychomotor retardation, hypertension, edema, dysuria, pollakiuria, previous urinary tract infections or enuresis. A renal ultrasound was requested, which reported inadequate cortico-medullary differentiation and renal sinus displaying a duplicated pyelocalyceal system in the right kidney with no other significant findings. Blood tests revealed normal serum creatinine (0.8 mg/dL), hypoalbuminemia (2.47 g/dL) and increased total cholesterol (499 mg/dL). Uric acid, calcium, phosphorus and magnesium serum levels were in normal range. A urine analysis showed increased isolated proteinuria (300 mg/dL).

**Figure 2:**
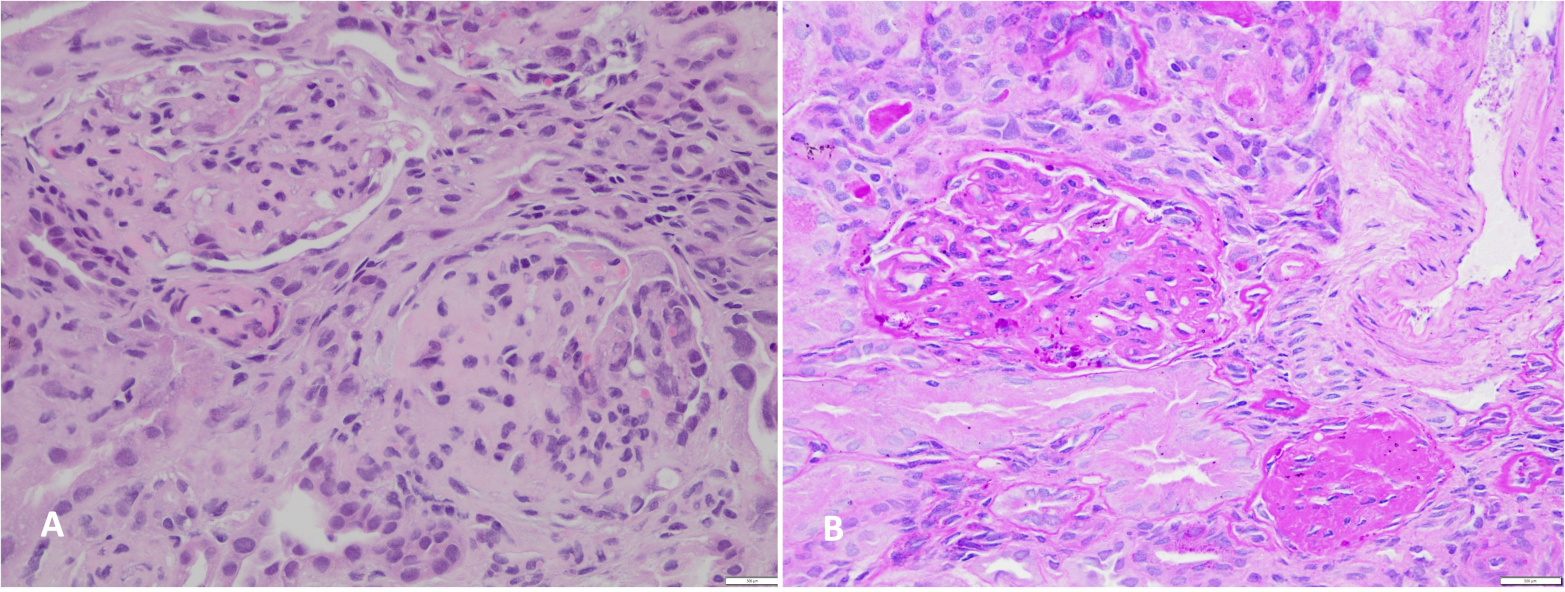
Histological findings in kidney biopsy revealed focal and segmental glomerulosclerosis. **(A)** Hematoxylin and eosin stain, 200x magnification. **(B)** Periodic acid-Schiff stain, 200x magnification.

The patient was reevaluated 7 months later, where the presence of edema became evident, with the following laboratory findings: hemoglobin 13 g/dL, hematocrit 34%, albuminemia 2.3 g/dL, serum creatinine 0.7 mg/dL, total cholesterol 579 mg/dL, urea nitrogen 34 mg/dL and a urine albumin to creatinine ratio of 4.0 mg/g. Oral prednisone treatment was initiated, but 4 weeks later no remission was observed. Bolus albumin was administered with no improvement of symptoms, leading to his hospitalization. Upon admission, the patient presented with urinary sodium <10 mEq/L, severe hypoalbuminemia (1.7 g/dL) and dry cough with clinical evidence of ascites and pleural effusion. Thoracentesis was performed to obtain pleural fluid for cytological analysis, which resulted negative for infections. Three boluses of albumin were administered with persistence of ascites and pleural effusion. Additionally, three intravenous boluses of methylprednisolone were given, with no changes in proteinuria. Due to the steroid-resistant behavior, a renal biopsy was performed, obtaining samples for light microscopy (LM), electron microscopy (EM), and immunofluorescence (IF). The biopsy specimen in LM comprised 18 glomeruli, with 8 of them globally and 2 segmentally sclerosed (**Figures 2A, B**). Additionally, there was moderate interstitial fibrosis and tubular atrophy. The examination performed by IF resulted negative after incubation with specific antibodies against heavy chains of IgA, IgG, and IgM, light chains Kappa and Lambda, complement factors C3c, C4, and C1q, as well as Albumin and Fibrinogen (**Supplementary Figure 1**). The sample submitted for EM did not contain glomeruli, limiting our ability to assess ultrastructural features in this specific case. Nevertheless, the combination of histopathological LM and IF features were considered compatible with FSGS and provided valuable insights into the structural alterations.

At this point, considering that remission remained elusive despite conventional therapeutic intervention, genetic analysis emerged as a critical diagnostic priority. The first genetic analysis was performed for *NPHS2*, considering that the p.R229Q and p.A284V variants in *NPHS2* are highly prevalent among Chilean patients with SRNS-FSGS (10). This genetic test was easily available but did not identify variants. Meanwhile, a protocol with cyclophosphamide was initiated, and oral prednisone dosage was reduced to 50 mg due to the high suspicion of SRNS. Whole Exome Sequencing (WES) identified a novel heterozygous variant of uncertain significance (VUS), *ACTN4* c.625_633del. This 9-bp deletion identified in exon 6 predicted an in-frame deletion of 3 amino acids, L209, I210 and E211, located within the ABD. Segregation analysis within the family was not possible and the *ACTN4* p.L209_E211del variant identified in our patient was assumed *de novo*.

**Figure 3:**
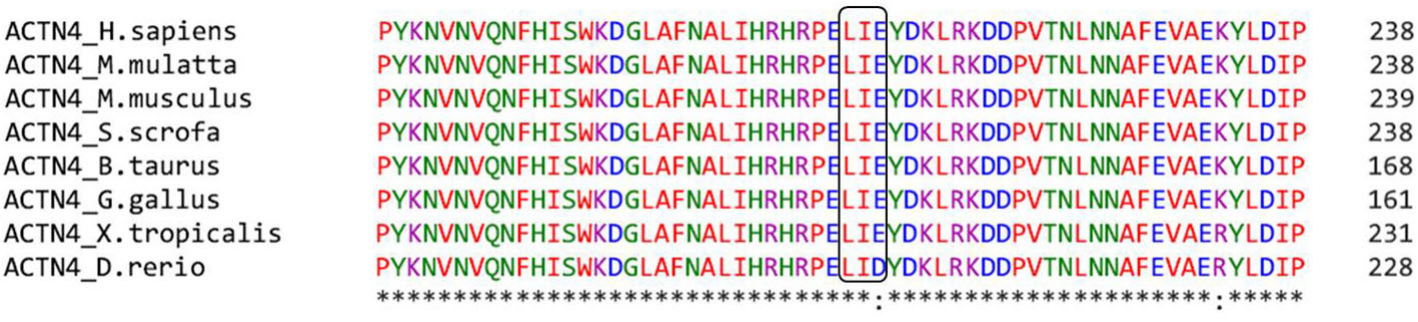
The sequence surrounding the position of the ACTN4 variant identified in the patient, was aligned with Homo sapiens (human), Macaco mulatta (monkey), Mus musculus (mouse), Sus scrofa (pig), Bos taurus (cow), Gallus gallus (chicken), Xenopus tropicalis (frog) and Danio rerio. The predicted deletion in *ACTN4* of the 3 amino acids, L209, I210 and E211, is highlighted in the black box.

Given a rapid progression to ESKD in less than 2 years, peritoneal dialysis was initiated. During this period, the patient presented an episode of reversible encephalopathy syndrome that evolved with a successful recovery. When the patient reached 14 years, he requested to be considered for kidney transplantation. Within this period a re-analysis of the VUS in *ACTN4* was performed. No registries of individuals carrying this variant were found in population databases (gnomAD, ESP and 1000 G). The analysis by a multiple sequence alignment program (Clustal Omega (https://www.ebi.ac.uk/Tools/msa/clustalo/) showed that the ACTN4 p.L209_E211del variant was located within the CH2 domain in a highly conserved sequence across vertebrate species (**Figure 3**). This suggests the critical importance of these amino acids and underscores their intolerance to substitution or elimination, thereby highlighting the functional significance and evolutionary conservation of this region. Until date, only one male patient with sporadic SRNS-FSGS had been identified carrying ACTN4 in-frame deletion of 3 amino acids (11). The variant involved Y260, V261 and S262 at the ending of the CH2 domain and was classified as likely pathogenic (PM1+PM2+PM4+PP3+PP4 criteria). Regarding our patient, the analysis by the metapredictor Varsome classified the ACTN4 p.L209_E211del variant as likely pathogenic (PM1+PM2+PM4 criteria). In addition, the heterozygous condition of the *ACTN4* variant resulted compatible with the autosomal dominant inheritance pattern described for SRNS-FSGS. Taking all this information into account, recurrence after transplant was considered to have a very low risk of recurrence. After a multidisciplinary evaluation, it was decided to enlist the patient in the national deceased donor waiting list.

Three months after enlistment, our patient received an allogeneic kidney allograft from a deceased donor, with a significant and progressive reduction of proteinuria within the first weeks. At his nephrological visit 5 months after transplantation, he presented serum creatinine 0.57 mg/dL, albumin 4.1 g/dL, and cholesterol 143 mg/dL. His creatinine clearance resulted in 101 mL/min, indicative of an optimal kidney function and a favorable prognosis.

## Discussion

In this case, incidental proteinuria was discovered in the context of a non-related pre-surgery analysis. This illustrates the importance of exhaustively studying and not ignoring these findings, especially in pediatric patients that start steroids in the context of proteinuric disease, since complications can be avoided with early diagnosis and prompt treatment (12).

Regarding the patient’s history, given the family circumstances, there is no perinatal information available, such as gestational age and birth weight, which would have been valuable during the case analysis. In addition, the presence of drug abuse during pregnancy is a known risk factor that cannot be ruled out as a phenotype modifier in our patient (13). Despite several atypical features during the initial nephrological evaluation, such as reduced kidney function, the absence of edema, and cryptorchidism, all highly suggestive of a genetic disease, genetic testing was not pursued at this time. Biopsy indication was made since the guidelines recommend that patients with SRNS undergo biopsy, except children with known or strongly suspected monogenic forms (14, 15) and the procedure is still considered the “gold standard” for the diagnostic evaluation of glomerular diseases (16). The biopsy results in our patient reported classical FSGS findings and the combination of LM and IF analysis provided valuable insights, prompting further genetic analysis to elucidate the specific molecular mechanisms underlying the clinical phenotype.

Considering resistance to first-line treatment, genetic analysis was initially conducted for NPHS2, as it is the most common genetic cause of SRNS in patients who present symptoms in late childhood. However, since the result was negative, a WES approach had to be performed. It identified a variant, *ACTN4* p.L209_E211del, located in the ABD, where most FSGS-associated variants are found. The variant was classified as VUS which could be explained mainly by the absence of registries of this specific variant in databases. Approximately two years later, in a report case published by He et al. (7), a novel heterozygous missense variant was found in the ABD in a 17-year-old Chinese girl, which motivated a literature review that reported 17 VUS and 22 pathogenic or likely pathogenic variants in the *ACTN4* gene. Notably, the majorities of the pathogenic or likely pathogenic variants were confirmed to be *de novo* and were located in the ABD between amino acids 50-269. This was a significant finding considering that the variant in our patient was a deletion of amino acids 209-211 in *ACTN4*, playing a fundamental role in the decision making process, because it motivated the re-analysis of the VUS, and consequently led to the consideration of kidney transplantation. After the surgery, the patient did not present recurrence during the subsequent 5 months, suggesting that the *ACTN4* p.L209_E211del variant, classified initially as VUS, was most likely the cause of the SRNS-FSGS.

The identification of VUS is a recurrent problem in routine clinical genetics, especially in patients with rare diseases or atypical phenotypes, who carry novel variants either through *de novo* occurrences or founder effects in populations with limited genomic resources. Recently, it has been noted that VUS variants make up the largest proportion of human genomic variations, comprising approximately 2 million entries in the ClinVar database (17). Rather than representing a dead end without further solutions, VUS should be re-analyzed as a standard of care in benefit of patients’ outcomes, considering the patient’s clinical evolution.

Within the last decade, an exponential growth in clinical genetics has been observed contributing to the ongoing development of bioinformatic tools for variant analysis, such as dynamic protein conformation, flexibility and stability predictors (19). Genetic testing is increasingly becoming accessible, even in countries with limited genomic resources. It has been suggested that the cost-effectiveness is notable when conducted during the early stages of specific kidney diseases, potentially resulting in significant cost savings, especially in pediatric cases (20). However, potential risks should always be assessed by a multidisciplinary team to balance risks and benefits that need to be communicated to the patients and their families. Our concern in the patient was the probability that he had an idiopathic non-genetic SRNS that in over 60% of cases showed a complicated clinical course after transplantation according to a recent systematic review and meta-analysis (18).

The use of exome sequencing to identify variants has demonstrated clinical utility, particularly in the context of rare diseases. Establishing a program for these conditions appears indispensable and feasible in countries with limited genomic resources (21). Its global adoption is foreseen to increase over time, provided that costs continue to decrease, and researchers and physicians enhance their training. Undoubtedly, more efforts are needed to foster research and to promote reaching a genetic diagnosis in patients, aligning with the goal to ‘leave no one behind’ as advocated by the World Health Organization and the United Nations, ideally through collaborative data-sharing initiatives.

## Data Availability

The dataset presented in the study are deposited in the FigShare repository, accession DOI number is https://doi.org/10.6084/m9.figshare.28212140.v1.

## Supplementary material

Supplementary Figure 1 Immunofluorescence analysis resulted negative for IgG, Kappa, and Lambda. IgA, IgM, and C3c were also negative (not shown).
