# Supplementary figures and images for: Case report: Novel ACTN4 variant of uncertain significance in a pediatric case of steroid-resistant nephrotic syndrome requesting kidney transplantation
Source: Front Nephrol. 2025 Jan 31;4:1375538. doi: 10.3389/fneph.2024.1375538 (PMC11826236; doi:10.3389/fneph.2024.1375538)

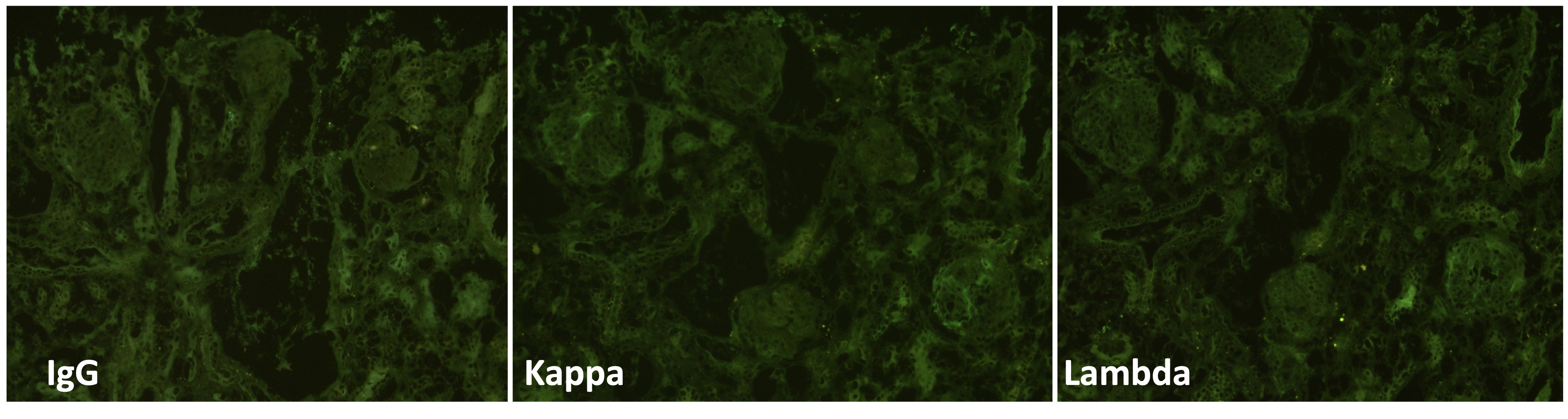

Supplement: Supplementary Figure 1 — Immunofluorescence analysis resulted negative for IgG, Kappa, and Lambda. IgA, IgM, and C3c were also negative (not shown). [file Image1.jpeg]
